# Application value of magnetic resonance imaging in diagnosing central nervous system lymphoma

**DOI:** 10.12669/pjms.322.9013

**Published:** 2016

**Authors:** Shanhua Zhang, Hongjun Li, Rongguang Zhu, Mingming Zhang

**Affiliations:** 1 Shanhua Zhang, Department of Radiology, Binzhou People’s Hospital, Binzhou, Shandong, 256610, China; 2 Hongjun Li, Department of Radiology, Binzhou People’s Hospital, Binzhou, Shandong, 256610, China; 3 Rongguang Zhu, Department of Radiology, Binzhou People’s Hospital, Binzhou, Shandong, 256610, China; 4 Mingming Zhang, Department of Radiology, Binzhou People’s Hospital, Binzhou, Shandong, 256610, China

**Keywords:** Magnetic resonance imaging, Central nervous system lymphoma, Primary central nervous system lymphoma, Imaging performance

## Abstract

**Objective:**

To describe the magnetic resonance imaging (MRI) appearance of central nervous system lymphoma.

**Methods:**

We retrospectively reviewed MRI images of 40 patients who had pathologically proven primary central nervous system lymphoma (PCNSL) and received treatment in Binzhou People’s Hospital, Shandong, China from January to December in 2014. Location, size and form of tumor was observed and relevant data were recorded for analysis.

**Results:**

Foci of 40 cases of PCNSL all located in brain, among which. 18 cases were single (45.0%) and 22 cases were multiple (55.5%). Of 96 Foci, 84 were supratentorial, 12 were subtentorial. Enhanced MRI scanning showed that, most Foci had significant homogenous enhancement, shaping as multiple nodular or lumpy, and few had ring-enhancement. MRI suggested that, T1 signal of most Foci concentrated on low signal segment and T2 signal gathered on high signal segment, suggesting a significant homogeneous enhancement; moreover, mild and medium edema surrounded the tumor. They were pathologically confirmed as B cell derived non-hodgkin lymphoma. Except one case of Burkitt lymphoma, the others were all diffuse large B cell lymphoma which was observed with diffuse distribution of cancer cells (little cytoplasm, large nucleus, rough perichromatin granule) in same size. Fifteen cases were observed with sleeve-like infiltration of cancer cells around blood vessels. No case was found with hemorrhage, necrosis or calcification.

**Conclusion:**

Pathological foundation of PCNSL determines its characteristic MRI performance. Typical case of PCNSL can be diagnosed accurately by MRI.

## INTRODUCTION

Primary central nervous system lymphoma (PCNSL) is a seldom seen non-Hodgkin’s lymphoma derived from brain, spinal cord and eye, accounting for 3% to 5% of intracranial tumors. 1% of all lymphoma only accounts for no more than 5% among all non-Hodgkin’s lymphoma.^1^,^2^ Recently, incidence of PCNSL has become increasingly higher among patients with or without abnormal immune. PCNSL is more likely to be misdiagnosed though there are many studies focusing on PCNSL in China and abroad.^3^,^4^ It is of great clinical significance to carry out preoperative imaging diagnosis as malignant lymphoma is sensitive to radiotherapy and chemotherapy and surgical operation cannot control PCNSL.

Magnetic Resonance Imaging (MRI), the major method used for diagnosing PCNSL currently, has outstanding advantages in localization diagnosis of intracranial tumours. It can not only clearly display intracranial anatomical structure, but can also precisely position.^5^,^6^ Primary lymphoma mostly starts from cerebral hemisphere, and 70% ~ 80% are supratentorial focuses locating in deep white matter, callosum, basal ganglia and thalamus, some of which also involves pia mater and ependyma.^7^,^8^ To improve recognition of PCNSL, this study collected 40 cases of pathologically proven PCNSL with complete MRI plan scan and enhancement scan materials for analysis of pathological analysis and discussing MRI characteristics of PCNSL.

## METHODS

### General data

In this study, 40 PCNSL patients who received treatment in Binzhou People’s Hospital, Shandong, China from February 2014 to December 2014 were taken as research subjects. Of 40 PCNSL cases, 28 were males and 12 were females, with an average age of (58.20±4.20) years. Clinical performance of patients included headache, vomit, lack in strength, visual field defect, fever, etc. Except for five cases, the others were mostly misdiagnosed as glioma, metastatic tumor, meningeoma and ependymoma before surgery. All cases have had no congenital or acquired immune defect previously. They have not treated with hormone before MRI examination and confirmed having no systematic lymphoma by examination of marrow cell and other examinations.

### Method

Signa 1.5T MAR scanner (GE) was used to perform MRI plain scan on head. Parameters were as follows: transverse view T1 Weighted Image (T1WI) (time of repetition (TE): 300 ~ 500 ms; time of echo (TE): 8 ~ 12 ms), Fast Spin Echo (FSE) (TR: 2500 ~ 5000 ms; TE 90 ~ 102 ms), fluid attenuated inversion recovery (FLAIR) (TR: 8002 ms; TE: 104 ms), echo-planar imaging - diffusion weighted imaging (EPI-DWI) (TR: 7100 ms; TE: 129 ms); sagittal view spin echo T1WI (TR: 300 ~ 500 ms; TE: 8 ~ 12 ms); coronal view FSE T2WI (TR: 2500 ~ 5000 ms; TE: 90 ~ 102 ms). Enhancement scan was also carried out. Parameters were: transverse view, sagittal view and coronal view SET1WI (TR: 300 ~ 500 ms; TE: 8 ~ 12 ms), Gd - DTPA (0.1 mmol/kg). Slice thickness was set as 6.0 mm, interval was 2.0 mm, field of view was 18.0 cm × 18.0 cm and matrix was 256×256.

### Image and pathological observation

All images were reviewed by two experienced neuroimaging doctors to observe MRI signal characteristics, location, form and size of tumor, boundary peritumoral edema, existence of necrosis, cystic change, haemorrhage and calcification, enhancement degree, mass effect and whether tumor involved ependyma and pia mater. T1 and T2 signal strength of lesion could be typed into low, slightly low, medium, slightly high and high. Peritumoral edema^2^ could be graded into mild edema (< 1/2 diameter of tumor), medium edema (> 1/2 diameter of tumor, but < diameter of tumor) and severe edema (> diameter of tumor). Total removal or subtotal removal of lesion was performed in craniotomy for nineteen cases; 4 cases were given sereotactic mamography biopsy. Tumor tissue was fixed by 10% neutral buffered formalin, embedded with paraffin, stained with hematoxylin and eosin (HE). Cell distribution density, nucleus to cytoplasm ratio, fibre composition as well as distribution and form of blood vessels of tumor tissue were observed. All cases were processed by immunohistochemical staining.

### Statistical method

SPSS version 19.0 software was applied for analyzing data. Measurement data were expressed as mean±SD. Counting variable in different groups was compared using chi-square test. Difference was considered to be statistically significant if P<0.05.

## RESULTS

### Distribution features of PCNSL and its MRI performance

Of 40 cases of PCNSL, 18 cases were single (45.0%) and the remaining 22 cases were multiple (55.5%). There were totally 96 focuses, involving 14 sites of body. Details are shown in Table-I.

**Table-I:** Distribution of 96 focuses.

Location of focus	No. of cases
Callosum	6
Below callosum	8
Central line of forehead	6
Central line of parietal lobe	6
Left occipital midline	6
Right occipital midline	6
Beside left encephalocoele	10
Left basal ganglia	8
Right basal ganglia	6
Surface of left temporal lobe	6
Surface of right temporal lobe	14
Left ependymal layer	6
Aqueduct of sylvius	8

**Fig.1:**
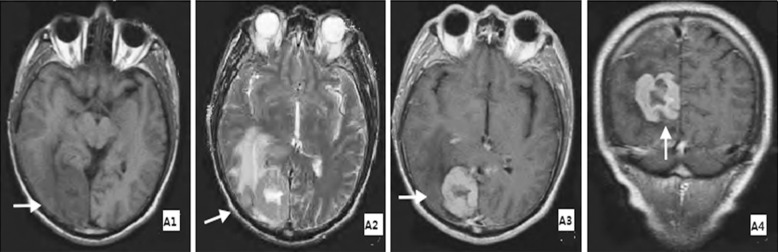
MRI performance for head of typical PCNSL patients. ***Note:*** A1: transverse view-T1WI, tumor with relatively low signal on right occipital lobe; A2: transverse view-T2WI, mass tumor with relatively high signal on right occipital lobe, medium edema around tumor; A3: transverse view-enhanced T1WI, significantly enhanced tumor with featured notch sign; A4: coronal view-enhanced T1WI, significantly enhanced tumor with featured notch sign. The place where the white arrow points is the location of tumor.

Of 96 foci, most was shaped as nodular or lumpy, significantly enhanced. Nodular focuses and mass focuses has characteristic notch sign (Fig.1) and pointed sign. As to MRI signal on T1WI, 76 had low signal (Fig.1-A1), 6 had medium signal, 10 had relatively high signal and 4 had high signal; as to MRI signal on T2WI, 24 had relatively low signal, 4 had medium signal, 64 had relatively high signal (Fig.1-A2) and 4 had high signal. Moreover, 78 foci showed up significant enhancement (Fig.1-A3 and Fig.1-A4), accounting for 81.20%. Mild-medium edema was found around tumor (Fig.1-A2).

### Analysis of MRI signal of PCNSL

PCNSL focuses showed homogeneously or inhomogeneously low signal on T1WI; most focuses on T2WI exhibited medium or relatively high signal and few showed high signal. Details are demonstrated in Table-II.

**Table-II:** Distribution of 60 focuses.

Location of focus	No. of cases
Callosum	8
Below callosum	4
Central line of forehead	2
Left occipital midline	4
Right occipital midline	4
Beside left encephalocoele	6
Left basal ganglia	6
Surface of left temporal lobe	4
Surface of right temporal lobe	6
Left ependymal layer	6
Aqueduct of sylvius	10

### Surgery and pathology

**Fig.2:**
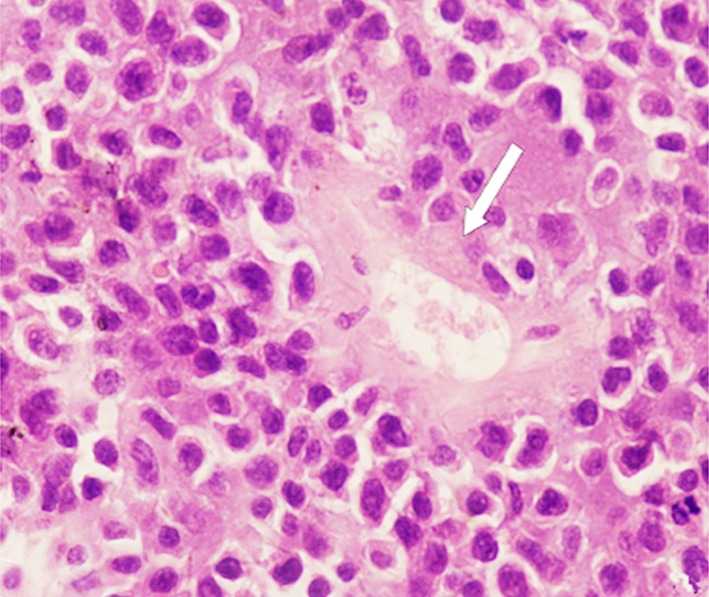
Female, 69 year-old (HE, ×200) ***Note:*** pathologically confirmed as diffuse large B cell lymphoma; intensively distributed tumor cells with little cytoplasm, large nucleus and rough perichromatin granule; sleeve-like infiltration of tumor cells around blood vessels (the position that white arrow points at).

**Fig.3:**
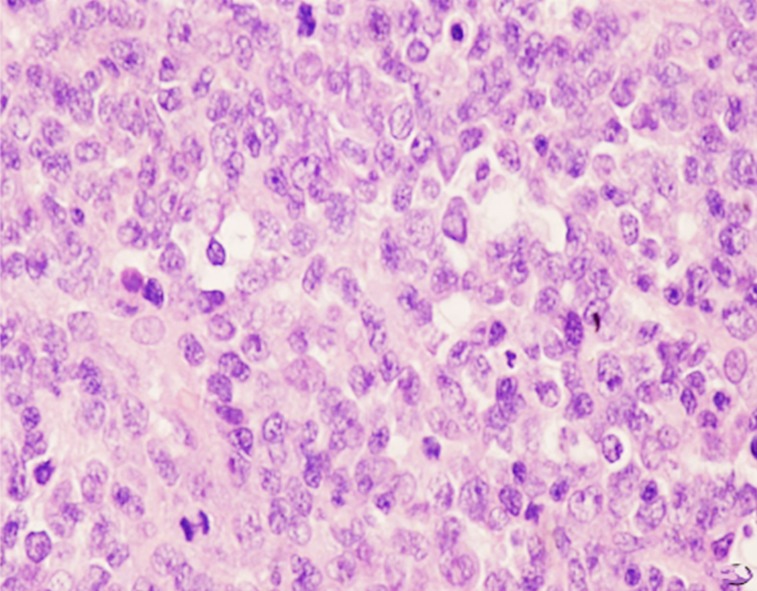
Male, 55 year-old (HE, ×200) ***Note:*** pathologically confimred as Burkkit lymphoma; intensively distributed cancer cells with obvious mitotic figure and sky star phenomena.

**Fig.4:**
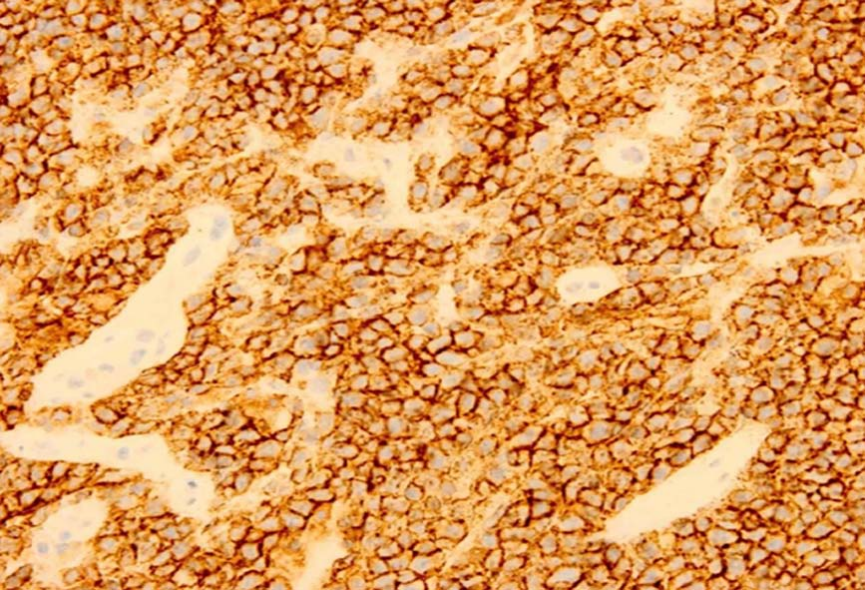
Female, 69 year-old (HE, ×200) ***Note:*** Pathologically confirmed as diffuse large B cell lymphoma; positive CD20; perivascular cuffing.

Tumor, in gray red or gray white color, was soft and without envelope, suggesting insufficient blood transport. Forty cases were all B cell derived non-Hodgkin’s lymphoma, including 38 cases of diffuse large B cell lymphoma and 2 cases of disperse Burkkit lymphoma. Diffuse large B cell lymphoma was observed with intensively distributed tumor cells (little cytoplasm, large nucleus, rough perichromatin granule) in same size. Fifteen cases were observed with sleeve-like infiltration of tumor cells around blood vessels (Fig.2); tumor tissues contained a small proportion of mesenchymal components. Besides the above characteristics, Burkkit lymphoma also showed obvious mitotic figure as well as sky star phenomena (Fig.3). Four cases were observed with cystic change, hence suggesting no obvious hemorrhage, patchy necrosis and calcification. Histochemical staining found positive leucocyte common antigen (LCA), positive CD20 (Fig.4) and positive Bc-16 as well as positive CD79a and CD10 in cases of Burkitt lymphoma.

## DISCUSSION

PCNSL, non-non-Hodgkin’s lymphoma only involving central nervous system, is mostly seen in brain parenchyma and meninx.^9^ PCNSL can occur to any age group, especially male aged from 20 to 50 years. PCNSL is more likely to be found in patients with transplanted organ or immunodeficiency abroad. Clinical and laboratory examination found no immunodeficiency in patients. Cause and pathogenesis of PCNSL occurring to people with normal immunity has not been known clearly.^10^ Clinical performance of PCNSL is non-specific, and its commonly seen clinical symptoms includes headache, emesis, epilepsy, focal dyskinesia and mental disturbance which are correlated to location and size of tumor.^11^,^12^ Patients who are definitely diagnosed having PCNSL is not suggested to undergo surgical treatment as PCNSL is highly sensitive to radioactive therapy.

A foreign study suggests that, PCNSL frequently occurs in cerebral hemisphere, callosum, basal ganglia and thalamus, less frequently occurs in epencephalon and brainstem and seldom occurs in ventricle only; 87% of PCNSL is supratentorial lesion. The findings are consistent with this study.^13^ Except for 12 subtentorial lesions, the remaining lesions were all supratentorial; most patients with normal immunity had single lesions; the largest lesion located at callosum; 18 single lesions (45%) located in cerebral hemisphere and most single lesions distributed in frontal lobe; multiple lesions locating on one side or both sides of cerebral hemisphere was featured by multi-center infiltration and closed to subarachnoid space, which is consistent with the findings of the study released by Coulon A et al.^14^ Patients with weak immunity or immunodeficiency, especially those who are infected with HIV are more likely to have multiple lesions that is easy to have cystic change, necrosis, hemorrhage or calcification and those lesions usually locate in deep of brain tissue.^15^,^16^

Histologically, high tumor cellularity, ratio of nucleus to cytoplasm and rich fibre composition composition of PCNSL determines its MRI signal characteristics, i.e., medium and slightly lower signal in T1WI and medium and slightly high signal in T2WI. Results of the study indicated that, PCNSL lesion exhibited medium or slightly low signal in T1WI and most lesions exhibited medium or slightly high signal in T2WI, which is consistent with research results of Johnson BA et al.^17^ Multiple lesions is featured by infiltration and some small lesions exhibited long T1 signal and T2 signal, which is attributable to the impact of surrounding edema on small lesion and small amount of cancer cells formed in the early stage of infiltration, MRI enhancement scan can qualitatively diagnose lesions. Fist-like or lumpy enhancement is frequently seen in people with normal immunity; whereas patients with immunodeficiency usually shows ring enhancement. In this study, most cases showed nodular or lumpy homogeneous enhancement and exhibited typical notch sign and pointed sign which is consistent with the description of Bessel EM et al.^18^ and is useful for qualitative diagnosis.

## CONCLUSION

PCNSL tends to occur more frequently and is more likely to grow in cerebral hemisphere, callosum or around ventricle. Pathological foundation of PCNSL determines its characteristic MRI performance. Most lesions showed medium or low signal in T1WI and medium or slightly low signal in T2WI. PCNSL cases were observed with fist-like, lumpy or nodular enhancement. Most PCNSL lesions can be accurately diagnosed using MRI technology and definite diagnosis relies on pathological examination.
